# Pediatric solitary pulmonary tuberculoma mimicking peripheral pulmonary hamartoma with extensive pleural adhesions: a case report

**DOI:** 10.3389/fmed.2025.1706112

**Published:** 2025-12-15

**Authors:** Kaiyi Mao, Xianhui Shang, Leibo Wang, Cao Wang, Yuchen Mao, Guangxu Zhou, Peng Zhao, Hongyang Tan, Zhen Luo, Hong Ma

**Affiliations:** 1 Department of Pediatric Surgery, Affiliated Hospital of Zunyi Medical University, Zunyi, China; 2 Department of Pediatric Surgery, Guizhou Children’s Hospital, Zunyi, China

**Keywords:** solitary pulmonary tuberculoma, pulmonary hamartoma, misdiagnosis, pleural adhesions, case report

## Abstract

This report describes a rare case of solitary pulmonary tuberculoma in a pediatric patient. A 13-year-old male presented with a progressively enlarging solitary nodule in the left lung, identified via chest computed tomography (CT). The patient had no obvious symptoms of tuberculosis and no known history of exposure. The tuberculin skin test was negative. The imaging findings strongly resembled a peripheral pulmonary hamartoma, leading to a preliminary diagnosis of pulmonary hamartoma with plans for video-assisted thoracoscopic surgery resection. Intraoperative findings revealed extensive and dense pleural adhesions, complicating both the identification of the lesion and surgical procedures. Postoperative pathology confirmed pulmonary tuberculoma. Additional diagnostic tests, including acid-fast bacillus staining, *Mycobacterium tuberculosis* polymerase chain reaction, and T-cell immunospot test for tuberculosis infection, all returned positive results. The patient recovered without complications and received standard anti-tuberculosis therapy. No recurrence was observed during a 12-month follow-up. Therefore, in cases of solitary pulmonary nodules in children, particularly in regions with high tuberculosis prevalence, pulmonary tuberculoma should be considered, even when imaging features are consistent with pulmonary hamartoma. It is recommended that tuberculosis screening be enhanced through a combination of interferon-gamma release assay and tuberculin skin test, with needle biopsy employed when necessary to improve diagnostic accuracy and prevent misdiagnosis. Additionally, for pediatric patients with pulmonary tuberculoma, the potential impact of tuberculous pleuritis should be considered preoperatively, and anti-tuberculosis therapy may be required before surgery to reduce operative risks.

## Introduction

Tuberculosis (TB) is a chronic infectious disease caused by *Mycobacterium tuberculosis* (MTB) infection in humans. According to the World Health Organization (WHO) Global Tuberculosis Report 2024, there were approximately 10.8 million new TB cases worldwide, of which about 1.3 million (12%) were newly diagnosed in children and adolescents (1). Pulmonary tuberculosis (PTB) is the predominant form of TB in children. In recent years, the emergence of multidrug-resistant TB and extensively drug-resistant TB has led to a rising incidence of pediatric pulmonary tuberculosis. Given its insidious onset, the relatively low bacterial load in children, and the lack of specific clinical symptoms, the diagnosis of pediatric pulmonary tuberculosis remains highly challenging.

Solitary pulmonary tuberculoma (SPT) is a special manifestation of pulmonary tuberculosis, which is relatively rare in pediatric patients. Most tuberculomas originate from secondary pulmonary tuberculosis lesions, while a few evolve from primary pulmonary tuberculosis lesions. The formation mechanisms are primarily classified into three types: (1) After the resolution of infiltrative pulmonary tuberculosis, caseous necrotic lesions are surrounded by proliferating fibrous tissue; (2) Obstruction of the bronchus draining a tuberculous cavity leads to the retention of caseous material, which fills the cavity; (3) Several small tuberculosis lesions merge into a single lesion (2). SPT is often confused with malignant pulmonary tumors (3), but presentations resembling pulmonary hamartomas are exceedingly rare, with no relevant reports available to date.

Here, we report a rare case. Chest CT of the pediatric patient revealed a solitary nodule in the left lung, which progressively increased in size but was not accompanied by obvious symptoms or signs. The imaging findings resembled those of a peripheral pulmonary hamartoma, and results of the tuberculin skin test (TST) and other examinations were negative. Intraoperative exploration revealed extensive and dense adhesions between the lung and the entire pleural cavity, with the lesion located within the lung. Following complete resection, pathological examination confirmed the diagnosis of pulmonary tuberculoma.

## Case presentation

**Figure 1:**
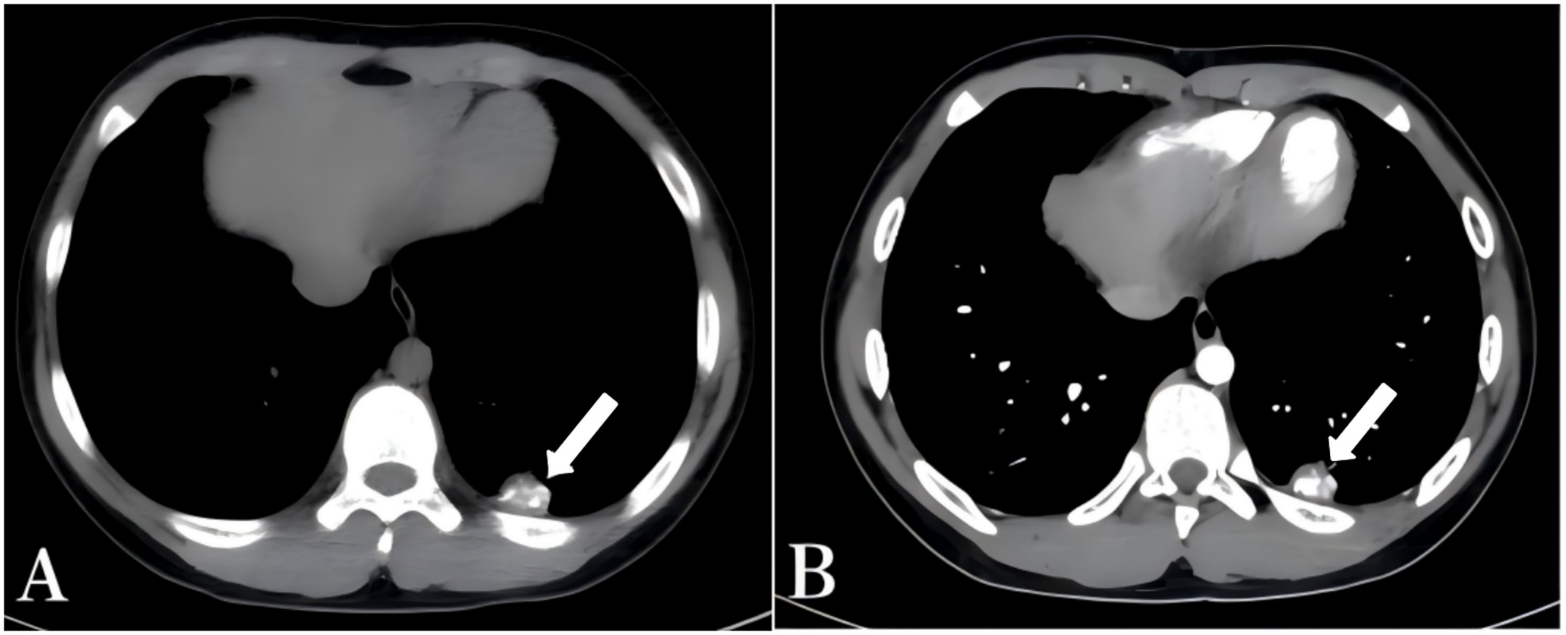
**(A)** Non-contrast CT reveals a mixed-density mass in the posterior basal segment of the lower lobe of the left lung, with a maximum cross-sectional dimension of approximately 23 mm × 18 mm. Multiple calcified foci are visible within the mass (arrow). **(B)** Contrast-enhanced CT shows heterogeneous enhancement of the lesion (arrow).

**Figure 2:**
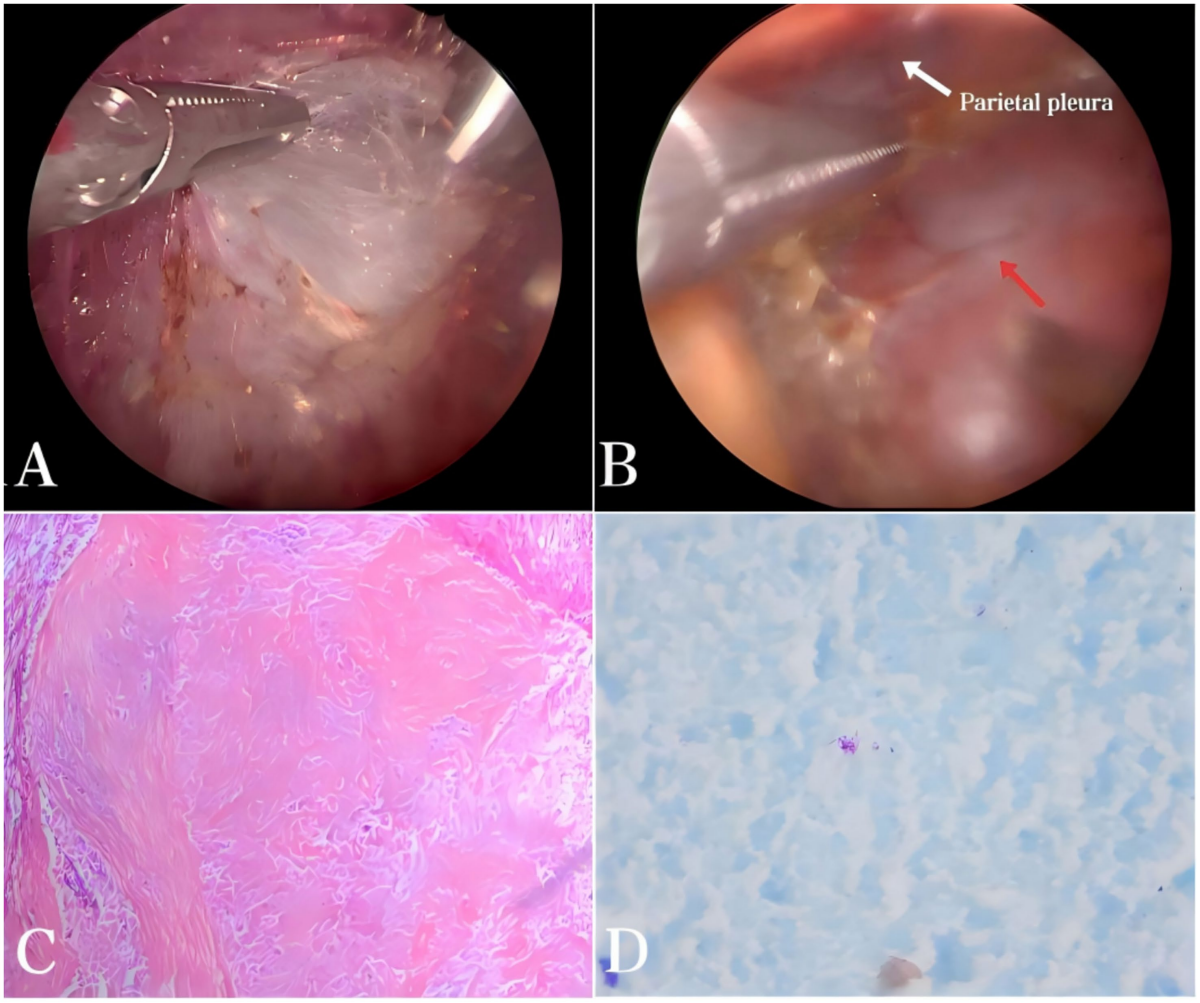
**(A)** Thoracoscopic examination revealed extensive loose fibrous adhesions within the pleural cavity. **(B)** A lesion approximately 25 mm × 20 mm in size was found in the posterior basal segment of the left lower lobe, with a firm texture (red arrow). **(C)** Histopathological examination (HE ×50) revealed a large, amorphous, eosinophilic, acellular necrotic region consistent with caseation, encased by dense fibrous tissue exhibiting focal hyalinization. **(D)** Acid-fast staining positive.

**Figure 3:**
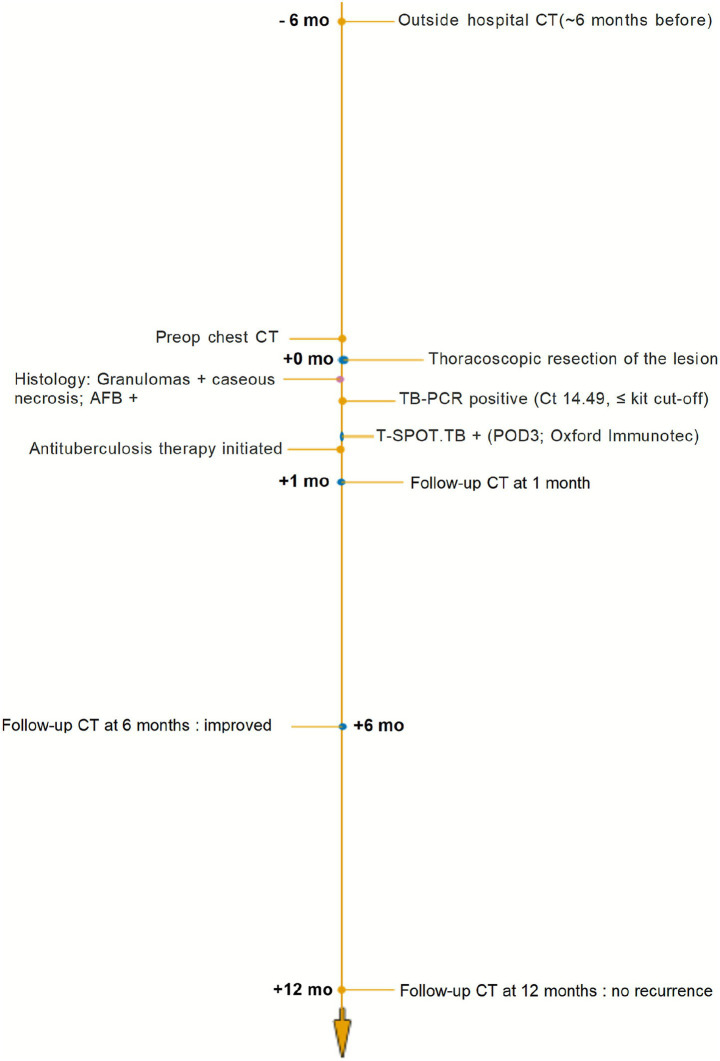
Chronological timeline.

A 13-year-old boy was admitted to our department due to a left thoracic space-occupying lesion that had gradually enlarged over six months. The patient had no chest pain, cough with sputum, hemoptysis, dyspnea, fever, or night sweats. His body habitus was normal, and physical examination revealed no abnormalities. Routine blood tests and erythrocyte sedimentation rate were within normal limits. Chest CT revealed a heterogeneous mass beneath the pleura in the posterior basal segment of the left lower lobe, with a maximum cross-sectional diameter of approximately 23 mm × 18 mm, containing multiple calcifications (Figure 1A). Contrast-enhanced CT demonstrated heterogeneous enhancement of the mass (Figure 1B). Previous imaging data were unavailable, and both the patient and his family denied a history of tuberculosis exposure. The purified protein derivative (PPD) skin test result was negative. Following multidisciplinary team discussion, the initial preoperative diagnosis was peripheral pulmonary hamartoma of the left lung, and thoracoscopic resection of the lesion was planned. Intraoperatively, extensive filamentous fibrous adhesions were observed in the left thoracic cavity (Figure 2A), resulting in injury to the lung lobe during port creation and difficulty in locating the lesion. After meticulous adhesiolysis, a firm lesion measuring approximately 25 mm × 20 mm was identified in the posterior basal segment of the left lower lobe (Figure 2B). The lesion was completely excised, and a closed thoracic drainage tube was placed. Postoperative histopathology revealed a large, amorphous, eosinophilic acellular necrotic area consistent with caseation, surrounded by dense fibrous encapsulation with focal hyalinization (Figure 2C). Acid-fast staining was positive (Figure 2D). Real-time PCR targeting the *Mycobacterium tuberculosis* IS6110 gene was conducted using a nucleic acid detection kit based on the PCR-fluorescent probe method (Beijing Boao Jingdian Co., Ltd.). A Ct value ≤25 was considered positive. In this case, the Ct value was 14.49, which was deemed positive. The final diagnosis was pulmonary tuberculoma. Peripheral blood was collected on postoperative day 3. The T-cell immunospot test for tuberculosis infection (T-SPOT.TB) assay (Oxford Immunotec Ltd., Abingdon, United Kingdom) was performed on freshly isolated PBMCs (2.5 × 10^5^ cells/well) stimulated with ESAT-6 and CFP-10 according to the manufacturer’s instructions. A positive result was defined as ≥6 spot-forming cells above the nil control. The patient’s result was positive. The patient recovered well, with removal of the chest drainage tube on postoperative day 3 and discharge on day 5, without complications such as pneumothorax or atelectasis. Following resection, the patient received antituberculosis therapy for 6 months: an intensive phase with isoniazid, rifampicin, pyrazinamide, and ethambutol for 2 months, followed by a continuation phase with isoniazid and rifampicin for 4 months. Doses were weight-based per pediatric standards. Adverse events were monitored monthly (clinical review; complete blood count; liver function), and no clinically significant toxicity occurred. Follow-up imaging was scheduled at 1–2 months after treatment initiation, then at 6 months and 12 months, showing no recurrence. A chronological timeline summarizes the clinical course (Figure 3).

## Discussion

Pulmonary tuberculoma is a rare manifestation of childhood pulmonary tuberculosis (4). SPT is primarily composed of caseous material encapsulated by a fibrous capsule, and its radiological appearance typically presents as a round or oval shadow, most commonly located in the apical-posterior segment of the upper lobe or the dorsal segment of the lower lobe. The lesion often contains punctate or patchy calcifications, with lobulated or spiculated margins, and is frequently accompanied by “satellite lesions” as well as adhesions to the adjacent pleura. In most cases, pulmonary tuberculomas show no significant enhancement on contrast-enhanced CT scans (5, 6). SPT is commonly misdiagnosed as peripheral lung cancer or inflammatory pseudotumor, but confusion with peripheral pulmonary hamartoma is exceedingly rare. Pulmonary hamartoma is a localized lesion formed by an abnormal mixture of cells and tissues, typically containing cartilage, epithelium, smooth muscle, and adipose components (7). It is often discovered incidentally and lacks specific clinical manifestations. On imaging, the presence of “popcorn-like” or “comma-shaped” calcifications or fatty components are considered characteristic features of pulmonary hamartomas (8).

In this case, the patient had no prior history of tuberculosis exposure and no symptoms of tuberculous intoxication. Both the erythrocyte sedimentation rate and the TST results were negative. The radiological findings closely resembled those of pulmonary hamartoma and lacked the typical spiculated margins and “satellite lesions” characteristic of pulmonary tuberculoma. Therefore, a preoperative diagnosis of peripheral pulmonary hamartoma was favored, which led to misdiagnosis. The lesion exhibited “popcorn-like” calcification, possibly due to long-term accumulation of caseous necrotic material in the center of the tuberculoma, resulting in the formation of massive calcified deposits. Consequently, the intralesional calcification no longer appeared as fine punctate or ring-like, but instead presented as large blocky or clumped calcifications.

Studies have shown that the TST has low sensitivity, leading to false-negative results in some patients infected with *Mycobacterium tuberculosis*, particularly among children, the elderly, individuals with compromised immunity, and those with malnutrition (9). T-SPOT.TB is one type of interferon-gamma release assay (IGRA), and the diagnostic value of IGRA in individuals with normal immune function has been well recognized. Compared with TST, IGRA demonstrates comparable or higher sensitivity and significantly greater specificity. As a result, an increasing number of guidelines have approved the use of IGRA for diagnosing latent tuberculosis infection (LTBI) in children (10). Some researchers believe that, given the challenges in diagnosing LTBI in children, the performance of IGRA may be a key factor in determining whether to initiate preventive treatment. When TST and IGRA are performed simultaneously, IGRA can help improve the accuracy of LTBI diagnosis in children, especially when the results of the two tests are inconsistent. Therefore, in high-risk pediatric populations, both IGRA and TST should be performed. If either or both tests yield positive results, the patient should be considered infected (11). In this case, the patient lacked specific clinical manifestations. Based on the imaging findings, the probability of tuberculosis was judged to be low preoperatively. Consequently, only the TST was performed, while the epidemiological background of the patient being from a high-burden tuberculosis area was overlooked, and no further tuberculosis-related tests were conducted. As a result, a preoperative diagnosis of peripheral pulmonary hamartoma was favored, ultimately leading to misdiagnosis. However, the postoperative T-SPOT.TB test result was positive, consistent with the findings of the aforementioned studies.

Pulmonary tuberculoma responds poorly to antituberculosis drug therapy because the dense fibrous capsule hinders effective drug penetration, usually requiring a prolonged treatment course. Video-assisted thoracoscopic surgery (VATS) has demonstrated significant efficacy in the treatment of pulmonary tuberculoma. It can shorten the duration and dosage of antituberculosis therapy and is also applicable to cases unresponsive to conventional medical treatment (12). Some studies suggest that preoperative antituberculosis therapy may not be necessary, and only routine postoperative treatment for 6 to 12 months is required (13). It is noteworthy that, in this case, extensive, dense, filamentous pleural adhesions were observed throughout the thoracic cavity. Despite the use of one-lung ventilation (OLV) anesthesia intraoperatively, severe adhesions prevented adequate collapse of the lung tissue. This led to partial lung injury during the creation of the surgical access port and limited exposure of the operative field, thereby increasing surgical risk. Previous reports on adult pulmonary tuberculoma have not described such extensive adhesions (14). In patients with tuberculous pleuritis, the release of multiple cytokines, combined with inflammation and hypercoagulability, stimulates pleural tissues, resulting in fibrin deposition, pleural adhesions, and pleural fibrosis (15). The patient’s preoperative CT showed no overt signs of pleuritis—no pleural effusion, no appreciable pleural thickening, and no strand-like adhesions were identified—yet dense, diffuse adhesions were encountered intraoperatively. This inconsistency may be due to children may harbor occult or pauci-symptomatic pleuritis capable of driving fibrin deposition and organization despite minimal imaging correlates, thereby increasing operative difficulty. Owing to their relative immunologic immaturity, children may mount an exaggerated pleural cytokine response even during subclinical or early tuberculous pleuritis, which further promotes fibrin deposition, organization, and subsequent pleural fibrosis. This report has several limitations. A significant limitation is the absence of preoperative mycobacterial culture and phenotypic drug susceptibility testing (DST). Tuberculosis was not clinically suspected before surgery based on the child’s preoperative symptoms, laboratory profile, and imaging, which is why culture and DST were not performed.

In conclusion, this study reports a rare case of atypical pediatric pulmonary tuberculoma. The patient had no clear history of tuberculosis exposure and no symptoms of tuberculous intoxication. Both erythrocyte sedimentation rate and TST test results were negative, while imaging findings closely resembled those of pulmonary hamartoma. These factors collectively contributed to a preoperative misdiagnosis. In addition, extensive pleural adhesions were present in the thoracic cavity, which increased the difficulty of establishing the surgical pathway and performing intraoperative procedures. Therefore, in cases of solitary pulmonary nodules in children, particularly in regions with high tuberculosis prevalence, pulmonary tuberculoma should be considered, even when imaging features are consistent with pulmonary hamartoma. It is recommended that tuberculosis screening be enhanced through a combination of IGRA and TST, with needle biopsy employed when necessary to improve diagnostic accuracy and prevent misdiagnosis. Additionally, for pediatric patients with pulmonary tuberculoma, the potential impact of tuberculous pleuritis should be considered preoperatively, and anti-tuberculosis therapy may be required before surgery to reduce operative risks.

## Data Availability

The original contributions presented in the study are included in the article/supplementary material, further inquiries can be directed to the corresponding author.
